# Inattention and Uncertainty in the Predictive Brain

**DOI:** 10.3389/fnrgo.2021.718699

**Published:** 2021-09-28

**Authors:** Tuomo Kujala, Otto Lappi

**Affiliations:** ^1^Cognitive Science, Faculty of Information Technology, University of Jyväskylä, Jyväskylä, Finland; ^2^Cognitive Science, Traffic Research Unit, Faculty of Arts, University of Helsinki, Helsinki, Finland

**Keywords:** driving, predictive processing, occlusion, computational modeling, appropriate uncertainty

## Abstract

Negative effects of inattention on task performance can be seen in many contexts of society and human behavior, such as traffic, work, and sports. In traffic, inattention is one of the most frequently cited causal factors in accidents. In order to identify inattention and mitigate its negative effects, there is a need for quantifying attentional demands of dynamic tasks, with a credible basis in cognitive modeling and neuroscience. Recent developments in cognitive science have led to theories of cognition suggesting that brains are an advanced prediction engine. The function of this prediction engine is to support perception and action by continuously matching incoming sensory input with top-down predictions of the input, generated by hierarchical models of the statistical regularities and causal relationships in the world. Based on the capacity of this predictive processing framework to explain various mental phenomena and neural data, we suggest it also provides a plausible theoretical and neural basis for modeling attentional demand and attentional capacity “in the wild” in terms of uncertainty and prediction error. We outline a predictive processing approach to the study of attentional demand and inattention in driving, based on neurologically-inspired theories of uncertainty processing and experimental research combining brain imaging, visual occlusion and computational modeling. A proper understanding of uncertainty processing would enable comparison of driver's uncertainty to a normative level of appropriate uncertainty, and thereby improve definition and detection of inattentive driving. This is the necessary first step toward applications such as attention monitoring systems for conventional and semi-automated driving.

## Introduction

*“The output of the system is easily measured, and easily understood, but it is extremely difficult to specify what the input is that results in the observed output.”*

– Senders et al., 1967

Appropriate allocation of attention is needed for successful performance in many contexts—work, traffic, education, and sports, among others. In traffic, driver distraction is considered as a contributing factor in many accidents (Née et al., 2019). Driver distraction is one form of inattention, referring to insufficient attention allocation to activities critical for safe driving due to diverting attention to unrelated activities (Regan et al., 2011). Inattention could be also caused by, for instance, mind wandering or fatigue (Walker and Trick, 2018). Superficially, the phenomenon seems straightforward: performance errors become more likely when attention is not allocated in accordance to task demand at the right time (Fuller, 2005; Regan et al., 2011). Look more deeply, and it's a bit more complicated than that.

First, after-the-fact explanations of accidents and errors being “caused by inattention” leave many questions unanswered. Kircher and Ahlström (2017) and Regan et al. (2011) raise the issue of hindsight bias: the driver failed to give way to a bicyclist when turning, a crash occurred, and therefore the driver was “not paying enough attention.” A causal theory, in contrast, requires that one be able to independently define (and measure) if an operator is attentive, whether or not this leads to a performance failure. Only then can one predict and causally explain performance by (in)attention.

Second, being “fully attentive all the time” is not a realistic goal for most people, and most of the time not necessary to achieve a high level of safety. The crash risk of an experienced driver is extremely small (e.g., 1.38 crashes/million km on urban collector roads and 0.94 crashes/million km on rural arterial roads in USA according to Forbes et al., 2012). Even if inattention is often found to be involved in a crash, the occurrence of inattention often does not lead to a crash: the vast majority of episodes of momentary inattention on the road do not lead to accident (Victor et al., 2015). Drivers are able to adapt attention between the driving task and other tasks (e.g., operating the radio, talking on the phone; Tivesten and Dozza, 2015) or adapt the driving task (e.g., speed, following distance) according to their attention level (for review see Young et al., 2007, see also Fuller, 2005; Pekkanen et al., 2017, 2018). Kircher and Ahlström (2017) call for a definition of the minimum attentional requirements of safe driving.

To arrive at such a definition, the nature of attention in driving performance (and other similar “real-world” tasks) needs to be understood, at a theoretical level, in sufficiently precise terms. Toward this end, we outline a predictive processing approach to the study of attentional demand and inattention in driving, based on neurologically-inspired theories of uncertainty processing in the human brain and experimental research combining brain imaging, visual occlusion, and computational modeling.

## Attention as Management of Cognitive Resources and Uncertainty

There is a general consensus that human information processing resources are limited. There are perceptual and structural constraints in the human information processing architecture. The field of view is limited, and gaze (overt attention) is sequentially deployed to one object or location at a time (Land, 2006). Short-term or working memory capacity is limited to a small number of items that can be kept in mind simultaneously (Cowan, 2016). There are different psychological views on how attention relates to these constraints, and if it is composed of a single serial resource or multiple parallel resources (Meyer and Kieras, 1997), but its limited capacity is not in serious dispute. We consider here inattention as a form of inappropriate allocation of this limited resource in space and time.

How much attention is appropriate, and when? How should the “amount” of attention be defined in the first place? We propose that this fundamental question can be most fruitfully approached from the point of view of the unifying theory of predictive processing (Clark, 2013, 2015; Friston, 2018). The key conceptual connection is to consider the deployment of attention as management of uncertainty (Feldman and Friston, 2010), and (in)approriate attention as (in)appropriate uncertainty. In this framework, complete certainty is an unattainable ideal, just as being “fully attentive all the time” is—but there is a rational way to optimally take into account uncertainty in observations and in internal models in one's beliefs and in one's actions. This (Bayesian inference) is the core of the predictive processing theory (Clark, 2013, 2015).

**Figure 1:**
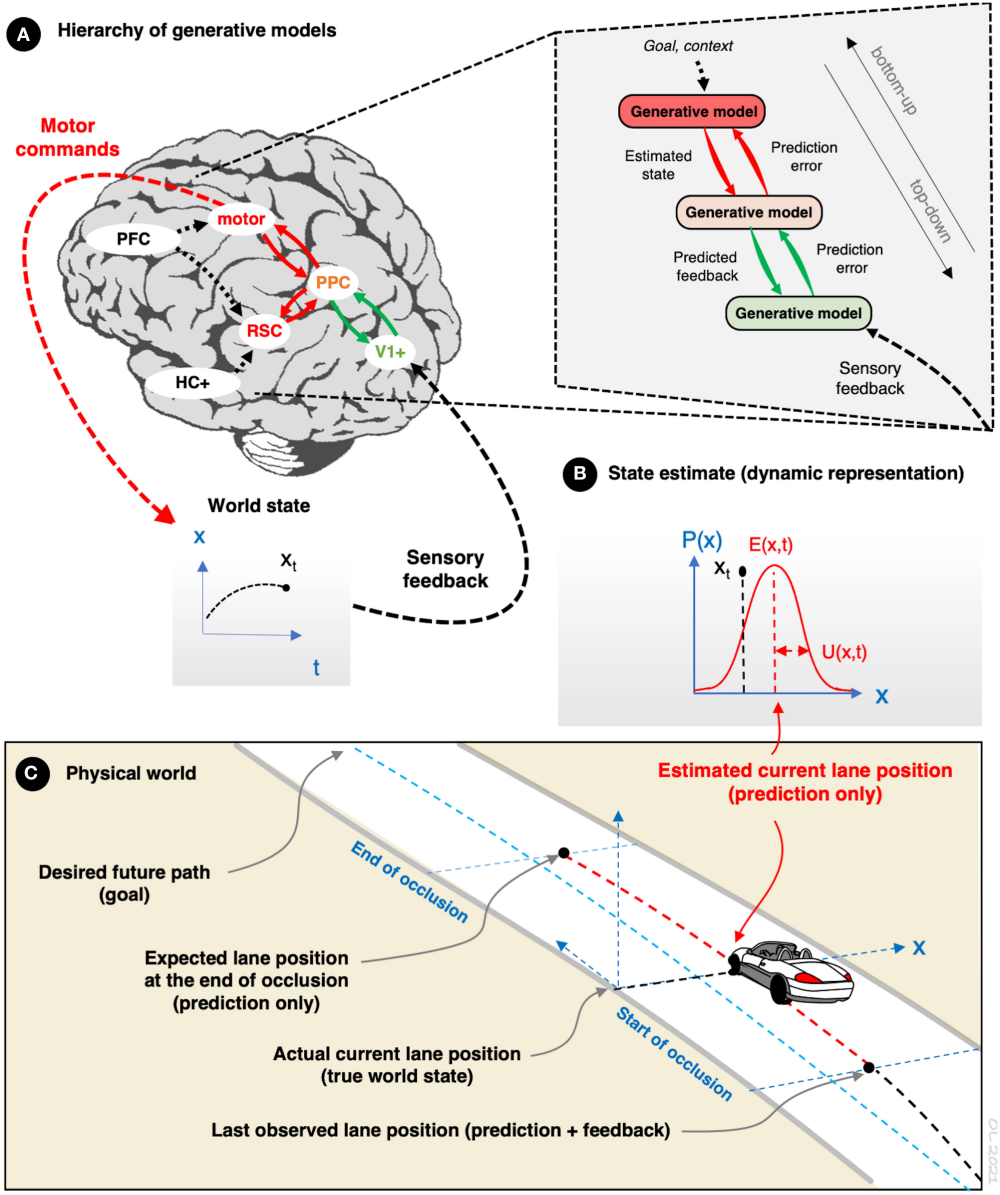
The predictive processing framework*. **(A)** Predictions are generated by a hierarchy of generative models. Information from sensory feedback is propagated bottom-up through the hierarchy by predictive coding, and learning is based on prediction error. At each level the internal models are trying to predict their own input (only), based on memory of past events and top-down context. At the bottom (sensory) level the predictions are directly about sensor observations. The a priori prediction is compared against feedback-updated estimates, and prediction error (only) is passed forward to the higher level. The progressively higher levels behave similarly, but more abstract features of the situation are predicted (complex perceptual features, objects, events, action outcomes). At each level, prediction error is used for learning to update the internal models to determine the a priori prediction at the next time step and for similar situations in the future. Crucially, the generative models and observations are always uncertain, but the system is assumed to know this and adapt to the uncertainty in an optimal (rational) way. **(B)** Variable x_t_ represents a world state x at time t, which is predicted, for example lateral road position in driving. At time t, a prediction of state x can be illustrated as a probability density function, where E(x,t) is the expected value of x and U(x,t) is a dispersion measure reflecting uncertainty of the expectation, such as variance. Note that the function does not have to be Gaussian or symmetric. **(C)** Illustration of car driving on a curved road under intermittent occlusion. While occluded (red line, e.g., during off-road glances, blinks, or saccades) the estimate of the state x is updated by top-down prediction only. Artificial occlusion methods allow the study of these predictions and the associated uncertainty estimates under controlled conditions. *Driving task relevant brain regions and functions in **(A)** (Navarro et al., 2018): PFC: prefrontal cortex (goals and task context; monitoring of task performance; also representation of uncertainty and connections to limbic reward system). Motor: pyramidal and extrapyramidal motor systems, premotor and supplementary motor cortex (coordination of motor responses). HC+: hippocampus and related structures like entorhinal cortex and parahippocampal gyrus (spatial context; also memory encoding and retrieval). PPC: (posterior) parietal cortex, precuneus (multisensory spatial attention; eye movements). V1+: visual cortex and associated subcortical regions (visual perception; visual scene analysis in parahippocampal and occipital place areas). RSC: retrosplenial cortex (connecting spatial, visual and multisensory attention systems).

Recent developments in cognitive science have led to suggestions that human cognition is just such an advanced prediction engine (Figures 1A,B; see Rao and Ballard, 1999; Friston, 2005, 2009, 2010, 2018; Hohwy, 2013; Clark, 2015). The function of this prediction engine is to support perception and action by continuously matching incoming sensory input against predictions of the input generated by a hierarchy of generative internal models representing statistical and causal regularities in the world. Prediction error is used as a learning signal to update the models. The generative models evolve iteratively by feedback (i.e., prediction error). The approach is based on well-understood concepts from signal processing theory and machine learning. Internal model update is Bayesian belief update for which computationally tractable approximations are known (e.g., for linear systems, the Kalmán filter), and for which plausible neurobiological implementations have been proposed (for review see Friston, 2010, 2018).

Based on the predictive processing theory (Clark, 2015), we will assume that the key cognitive functions of attention in this framework are to:

1. control memory recall, that is, the generation of top-down predictions to match against perceptual feedback, and
2. direct the active sampling of perceptual information, that is, bottom-up prediction error that reduces uncertainty about the situation (e.g., through eye-movements).

Attention may also affect, for example, how internal models are updated (given that this requires cognitive resources—an engaged driver will learn more and faster), and have many other functions we do not consider further here.

The core idea of predictive processing is that the brain takes into account the uncertainties of its own models and the incoming sensory information, and tries to strike an optimal balance between these two sources of information, the top-down and the bottom-up. Here, we propose that attention can be understood in terms of this balancing process and inattention as inappropriate balance, from some normative perspective, such as over- or underconfidence in one's predictions in relation to environmental volatility in traffic. Accordingly, appropriate attention can be recast as reflecting appropriate uncertainty about the situation and its potential outcomes^1^.

Our empirical approach to appropriate uncertainty builds on seminal work by Senders (1964); Senders et al. (1967), Sheridan's work (Sheridan, 1970) on supervisor's optimal sampling models and paradigms based on the visual occlusion technique (see esp. Kujala et al., 2016; Pekkanen et al., 2017). In visual occlusion experiments, the driver's sight is intermittently blocked by an occlusion visor, opaque glasses or screen on the windshield, or simply by blanking a driving simulator display (see Figure 1C). The driver can request a visual sample by pressing a button. Occlusion time and/or distance are calculated as the driver's estimate of spare visual capacity in driving (Safford, 1971). There is a lot of data on gross effects of various factors, such as road environment, road curvature, traffic, manoeuvre, age, and driving experience on spare visual capacity in driving (for review see Kujala et al., 2021) but a lack of a detailed understanding of the mechanisms behind these effects.

Occlusion scenarios admittedly lack some ecological face validity (information sampling in real driving is not through all or nothing occlusions), and perhaps for this reason have been less used in driver attention research than eye tracking (Kujala et al., 2021). But from the point of view of attentional processes—and especially computational modeling—the benefit is that it is not necessary to know how much and what kind of information is perceived and processed from the visual periphery (Pekkanen et al., 2017, 2018; Kircher et al., 2019; Kujala et al., 2021). Self-paced occlusion methods, in combination with other methods, allow more direct study of the predictions and the associated uncertainty estimates of the brain in controlled conditions. Note also that in natural driving, brief anticipated “occlusions” of a up to hundreds of milliseconds do occur up to several times a second (saccades, eye blinks, Land, 2006). Further, occlusion could be seen as mimicking multitasking while driving. The difference between true multitasking and occlusion on a single task is in that one can still fully focus (mentally) on the single task while occluded. Of interest could be to study the effects of additional tasks on the mental processes required for appropriate allocation of attention to the occluded task (Kujala et al., 2021).

## Appropriate Uncertainty in Predictive Processing

**Figure 2:**
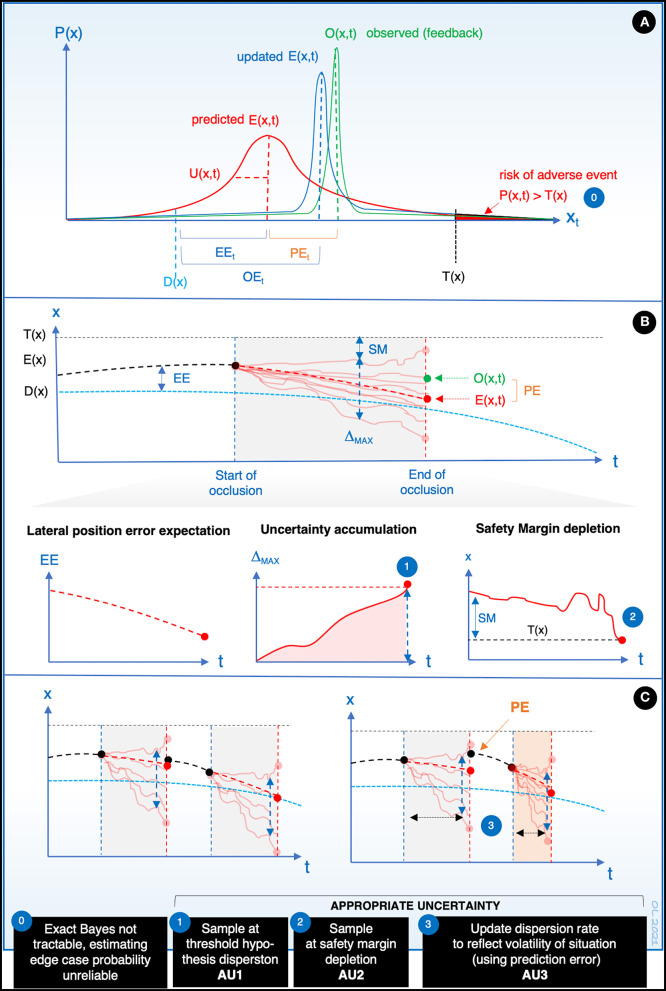
(Approximately) Bayesian inference in occluded driving, and three ways to understand appropriate uncertainty in the predictive processing framework (AU1-3). **(A)** At the end of occlusion (t, see Figure 1C), state information about x is updated by combining the internal prediction (red probability density function) and observed feedback (green) to yield the posterior (blue). E(x,t) is the expected value of the prediction, “expectancy,” and we refer to its difference from O(x,t), PE, as prediction error. D(x), desired x at time t (goal state); EE_t_, expected error at time t (difference of expectancy and goal state); OE_t_, observed error at time t. T(x), threshold × for subjective “failure” at time t (e.g., lane position where a wheel crosses a road edge), U(x,t) is a dispersion measure of the prediction distribution (here, standard deviation). Note that the functions do not have to be Gaussian or symmetric. **(B)** Illustration of the dynamic development of expectancy, expected error, uncertainty accumulation, and safety margin depletion, during an occlusion (see Figure 1C). This is conceived as trajectories of particles representing different hypotheses about the state that can be considered as a sample from the prediction distribution. Note that at the beginning of the occlusion, particle trajectories begin to diverge, corresponding to an increase of dispersion in the prediction distribution. Δ_MAX_= difference in predicted x between the most extreme hypotheses (the most extreme “subjectively possible” values of x). SM, safety margin, i.e., the distance from T(x) of the most extreme hypothesis. The occlusion ends (the driver requests a sample) when some criterion is reached, such as some value of prediction distribution dispersion or the depletion of safety margin. (Note that a sample will be requested even though the expectancy approaches the goal, i.e., expected error is reduced during the occlusion). **(C)** (Appropriate) uncertainty adjustment: observation of a higher PE at the end of the previous occlusion (right) leads to higher dispersion rate in the following occlusion, and hence more frequent sampling (AU3). This is an adaptive response to situational volatility signalled by PE.

Within the current framework, definition of appropriate uncertainty can be approached from at least three perspectives, each illustrated in Figure 2. In the example driving task (see Figures 1C, 2), state x is car's lateral position. The driver has two goals: (1) D(x) is the steering goal (i.e., desired path of the car) and (2) to keep the uncertainty of lateral position U(x) under a preferred constant is the sampling “goal.” D(x) also includes the implicit goal to stay on the road by remaining between road edges. A road edge defines here a task-critical threshold T(x) and (partly) a critical safety margin for the driver in the task.

Figure 2A shows an imaginary example of how, at the end of an occlusion, the driver's brain updates a prior prediction distribution about state x (car's lateral position) to a posterior distribution, based on observed feedback. It is assumed that this update is based on Approximately Bayesian Computation (the exact Bayesian distributions being intractable), that can be modeled with existing techniques such as particle filters. Note that for this reason estimation of the exact probability (or risk) of a very rare adverse event at observation, that is, x exceeding some task-critical threshold T(x) (e.g., road edge), can be highly unreliable for the brain [Figure 2A, (0)].

Figure 2B shows the dynamic development of expectancy, expected error, uncertainty accumulation and safety margin depletion, during an occlusion (cf. Figure 1C), that is, during a time interval while the driver is not observing state x. During the occlusion, the brain generates predictions, that is, samples hypotheses from the generative models, maintaining a dynamic prediction distribution about the development of state x in time.

We assume that the more hypotheses (models) sampled, the more attentive the driver is (cf. attention as control of memory recall). Paradoxically, this can mean that the more attentive, the faster the driver becomes uncertain of the development of state x during occlusion. For example, suppose the driver wishes to maintain occlusion until it is “possible” that a critical safety margin is breached [diffusion to a barrier; Figure 2B, (2)]. The more hypotheses, the faster the dispersion rate of the extreme values in the distribution, and therefore, the sooner the possible safety margin depletion under occlusion. This means that a more “attentive” driver will sample more frequently.

Here, it is important to notice that the expected prediction error [i.e., desired D(x) – predicted E(x,t)] can decrease in time (e.g., due to steering toward desired position) during the whole occlusion, but still uncertainty (e.g., the difference in predicted x between the most extreme hypotheses) will increase. Again, suppose the driver wishes to sample when some critical dispersion is “possibly” reached [Figure 2B, (1)]: the attentive driver will sample more frequently even if the driver “expects” the error at the end of occlusion to be small.

Furthermore, based on observed feedback (prediction error), attentional control of top-down processes (sampling the generative models) should adapt the number of hypotheses and their dispersion rate to be appropriately “calibrated” to the volatility of the situation, for future occlusions in similar situations. That is, a “big surprise” at the end of occlusion should lead to more frequent visual sampling [Figure 2C, (3)]. This is yet another form of appropriate uncertainty. Higher volatility (as signaled by prediction error) means more unpredictable behaviour of the predicted state due to, for instance, increase in speed, variable curvature or reduced friction on the road. Higher volatility should increase uncertainty of the associated predictions (i.e., higher number of possible hypotheses in our approach). If the driver is not reactive to increased prediction error (i.e., does not adjust the uncertainty appropriately), this could lead to overconfidence in predictions, actions based on highly inaccurate state estimates and overlong occlusions, with possible negative consequences for task performance.

Figure 2 illustrates these three approaches for defining appropriate uncertainty in the predictive processing framework. First, it is rational and appropriate uncertainty (AU1), to sample feedback of x at a subjective threshold of “maximum tolerated uncertainty” U(x), provided that the threshold is appropriate for the situation, and the accumulation of uncertainty itself is appropriately calibrated. Second, it is appropriate uncertainty (AU2) to sample at a personal safety margin threshold T(x), when it is merely “subjectively possible” that the threshold is breached—regardless of the expected E(x,t) or the probability of the event (which for edge cases may be too small to estimate reliably). Third, it is appropriate uncertainty (AU3) to increase the number of hypotheses sampled from the prediction distribution, and thereby increase the dispersion rate^2^ of the most extreme hypotheses and the uncertainty [U(x,t)] growth rate for a following occlusion, if the prediction error is large at the end of the previous occlusion. The driver is adapting uncertainty and thereby visual sampling on the basis of the size of the prediction error, which informs about the volatility of the situation (i.e., “uncertainty in the world”). This adjustment of dispersion rate of the hypotheses works also in the other direction; with repeated low prediction error, it is appropriate to decrease the number of the hypotheses and thereby eliminate farthest hypotheses and increase the occlusion time.

## Conclusions

We have introduced a definition of attention as appropriate uncertainty (and inattention as inappropriate uncertainty) in predictive processing, with an application to driving under conditions of intermittent visual sampling. The novelty here is the emphasis on internal uncertainty as the basis of appropriate attention (as opposed to the false ideal of “complete certainty”) and the balance between uncertainty growth rate “in the world” (i.e., volatility) and in the brain.

We have identified three criteria of appropriate uncertainty; (1) sampling perceptual feedback of state x at a personal threshold of maximum tolerated uncertainty (dispersion of predictions), (2) sampling at a personal safety margin threshold (most extreme prediction), and (3) increasing the uncertainty growth rate for a following occlusion (and for similar future situations), when the sampled prediction error is large. Violation against any of these rational behaviours can be seen as inappropriate uncertainty, and inattention (or excessive attention) toward state x.

Intuitively, the idea is that the uncertainty of, for instance, a car driver, should rise at an appropriate time and it should either grow or decrease appropriately based on changes in situational factors, such as one's own speed, relative speeds, and positions and behaviours of surrounding vehicles. It is not irrational to tolerate some uncertainty (or “risk”), which is unavoidable.

This definition suggests that “being attentive” does not mean that you are constantly processing as much task-relevant information as you possibly can, but that you are processing it to a sufficient degree to succeed in the task, based on your personal goals, previous experiences and while being sensitive to changes in environmental volatility (signaled by prediction error). Attentiveness is also not only about fixating something foveally but about processing the information and making appropriate adjustments to the uncertainty of predictions. In this framework, both overconfidence (too little uncertainty) and underconfidence (too much uncertainty) are suboptimal for the performance of a human operator (cf. Engström et al., 2018).

If the brain is indeed “Bayesian,” then these sorts of processes should be the core function of the brain (Clark, 2015; Friston, 2018). That is, if the predictive processing approach holds water, then handling uncertainty and prediction error characterizes operations at all levels of neural sensory and motor hierarchies. Brain imaging research on decision making under risk and uncertainty (often under the umbrella term “neuroeconomics”) has begun to reveal some specific brain structures that may play a central role in the representation of uncertainty (risk, volatility). These relate especially to the monoamine systems (norepinephrine and dopamine) and limbic structures such as the amygdala and the cingulate and orbitofrontal cortices (e.g., Angela and Dayan, 2005: acetylcholine and norepinephrine signals, Doya, 2008: norepinephrine and the orbitofrontal cortex, Rushworth and Behrens, 2008: prefrontal and cingulate cortex, Payzan-LeNestour et al., 2013: multiple distinct cortical areas and the locus coeruleus, Gordon et al., 2017: signal-to-noise-ratio in semantic wavelet induced frequency tagging, SWIFT). How this research relates to the neural substrates of driving (for review see Lappi, 2015; Navarro et al., 2018) remains an open question beyond the scope of this paper. However, from the predictive processing point of view the prediction would be that the hierarchy of networks sketched in Figure 1A (as identified in the meta-analysis of Navarro et al., 2018) would be a hierarchy of (top-down) predictions and (bottom-up) prediction errors. There are also uncertainty-based approaches to modeling cognitive processes that are not based on the predictive processing theories (e.g., Renninger et al., 2005; Vilares and Kording, 2011; Meyniel and Dehaene, 2017) but which might be compatible with the current approach.

Our approach introduces testable assumptions, hypotheses and novel research questions. We assumed that following prediction error the brain allocates attention (i.e., cognitive capacity) during occlusion by increasing sampling of hypotheses from the prediction distribution. Alternatively, the brain could choose to sample feedback (i.e., remove occlusion) at lower dispersion (i.e., at lower uncertainty threshold). Increased number of hypotheses with decreased occlusion time should become visible in neural correlates associated with processing of the hypotheses (cf. N1: Näätänen, 1992; P3b: Polich, 2007). Experimental designs that utilize additional tasks during occlusion could reveal how the additional tasks affect the mental processes of, for instance, hypothesis generation for the occluded task, and thereby, adjustments of uncertainty. However, the question to what extent “cognitive load” from secondary tasks relies on the same cognitive capacity as the primary (driving) task is a problem that is not yet well-understood. Besides multitasking, the effects of, for instance, cumulating driving experience, mind wandering and fatigue on uncertainty adjustment ability should be studied (and modeled). The most fundamental prediction from the theoretical approach is that when a driver is appropriately attentive toward a task-relevant state x, the size of prediction error at observation of x as detected from its neural correlates (e.g., Angela and Dayan, 2005: norepinephrine signals, Payzan-LeNestour et al., 2013: the locus coeruleus) should correlate with the following change in the sampling rate of the state (e.g., glancing frequency).

We believe that this kind of approach—combining a theoretical approach based on solid modeling concepts with a plausible physiological basis with a careful and accurate measurement and analysis of ecologically representative situations—has the potential to take the study of cognition and the brain out of the laboratory, and to address “real world” problems. These include, but are not limited to, ergonomics, human performance, attention monitoring, and safety in manual and automated driving. The approach is applicable to tasks and scenarios beyond lane keeping—and driving. For instance, driver's longitudinal control in a car following task (where x = safety distance or time-to-collision) can be computationally modeled, and has actually been modeled, as management of uncertainty (Johnson et al., 2014; Pekkanen et al., 2018).

Potential future applications of the proposed research approach include driver attention monitoring systems for conventional and semi-automated driving (Lenné et al., 2020). A proper understanding of uncertainty processing in the brain could enable comparison of driver's uncertainty to a normative level of appropriate uncertainty, and thereby improve the definition and detection of inattentive driving. However, the normative criterion for appropriate uncertainty must make theoretical sense, and it has to be well-defined. The outlined approach holds promise for delivering such a definition.

## Data Availability Statement

The original contributions presented in the study are included in the article/supplementary material, further inquiries can be directed to the corresponding author/s.

## Author Contributions

The original idea for the paper and the term appropriate uncertainty came from TK. All authors contributed equally to the conception of the paper, development of the argument, and writing of the final version.

## Funding

The research was partly funded by Academy of Finland (Sense of Space/334192 and Appropriate Uncertainty in Manual and Automated Driving/343259).

## Conflict of Interest

The authors declare that the research was conducted in the absence of any commercial or financial relationships that could be construed as a potential conflict of interest.

## Publisher's Note

All claims expressed in this article are solely those of the authors and do not necessarily represent those of their affiliated organizations, or those of the publisher, the editors and the reviewers. Any product that may be evaluated in this article, or claim that may be made by its manufacturer, is not guaranteed or endorsed by the publisher.

## Footnotes

^1^This concept is similar to Lee and See (2004) “appropriate trust' or “appropriate reliance.” However, uncertainty is more precise to define, and development of uncertainty easier to model computationally than trust, which is a phenomenological, much more subjective construct.

^2^Here, we assume that the brain adjusts the dispersion rate by increasing sampling of hypotheses from the prediction distribution. Another possibility is that the number of hypotheses and the dispersion rate stays at the same level but the brain lowers the threshold of maximum tolerated uncertainty, i.e., samples feedback at lower dispersion.
